# How Costs Influence Decision Values for Mixed Outcomes

**DOI:** 10.3389/fnins.2012.00146

**Published:** 2012-10-26

**Authors:** Deborah Talmi, Alex Pine

**Affiliations:** ^1^University of ManchesterManchester, UK; ^2^Department of Neurobiology, Weizmann Institute of ScienceRehovot, Israel

**Keywords:** cost-benefit analysis, decision-making, decision-making and neuroeconomics, economic models, reward, punishment, aversive decision-making

## Abstract

The things that we hold dearest often require a sacrifice, as epitomized in the maxim “no pain, no gain.” But how is the subjective value of outcomes established when they consist of mixtures of costs and benefits? We describe theoretical models for the integration of costs and benefits into a single value, drawing on both the economic and the empirical literatures, with the goal of rendering them accessible to the neuroscience community. We propose two key assays that go beyond goodness of fit for deciding between the dominant additive model and four varieties of interactive models. First, how they model decisions between costs when reward is not on offer; and second, whether they predict changes in reward sensitivity when costs are added to outcomes, and in what direction. We provide a selective review of relevant neurobiological work from a computational perspective, focusing on those studies that illuminate the underlying valuation mechanisms. Cognitive neuroscience has great potential to decide which of the theoretical models is actually employed by our brains, but empirical work has yet to fully embrace this challenge. We hope that future research improves our understanding of how our brain decides whether mixed outcomes are worthwhile.

When faced with many possible courses of action humans and animals must evaluate their expected future costs and benefits in order to decide optimally. Every action is associated with a cost because every action requires, at minimum, some energy expenditure for execution. The things that we hold dearest often require a sacrifice, as epitomized in the maxim “no pain, no gain.” We struggle to be included in our peer group, study hard to increase our career prospects, work to provide for our families, pay to go on vacation, subject ourselves to painful health tests to maintain our physical well-being, and spend considerable energy on caring for our loved ones. Understanding how costs are integrated with benefits to ultimately reach a decision is therefore of paramount importance.

Value-based theories of decision-making suggest that people are thought to evaluate courses of action on the basis of their predictions about the future happiness a choice will engender (Von Neumann and Morgenstern, 1947; Vlaev et al., 2011). Because people are notoriously bad at predicting their future emotions (Hsee and Hastie, 2006) their decision utility is often different from their experienced utility at the time the consequences of their action come to fruition (Kahneman et al., 1997). Here we focus on decision utility, the time when agents decide between different prospects. Our question is how the subjective value of outcomes that are mixtures of costs and benefits is established.

We define costs and benefits as outcome attributes that decrease or increase, respectively, the decision value of that outcome at the time of decision-making. The costs and benefits most often studied in cognitive neuroscience include primary reinforcers such as food, drink, physical effort, and pain; secondary reinforcers such as monetary gains and losses; and mental events such as cognitive effort (Kool et al., 2010) and emotional suffering, such as the pain of regret (Bell, 1982). Although in some situations effort may be rewarding (Kivetz, 2003; Kim and Labroo, 2011), it is normally considered a cost (Hull, 1943).

While the definition of benefits is straightforward, our definition of costs may be controversial because it excludes some aversive outcome attributes. For instance, a decision may be risky because it entails a chance that a reward is not obtained, or it may prolong the time until reward is available for consumption. Yet we do not consider risk and delay to be true costs because they do not produce a negative subjective utility on their own, in the absence of other rewards or costs. Both risk and delay derive their meaning from the nature of the outcome and modulate its utility; their emotional valence depends on whether the outcome is rewarding or costly (see Loewenstein, 1987, for discussion on the valence of delay). As we will see, all available models of value integration make a similar distinction in that they treat risk and delay differently to “true” costs.

There are several theoretical valuation models for integrating costs and benefits. Cognitive neuroscience has great potential to decide which one is actually employed by the brain. Yet the burgeoning behavioral and neurobiological work on decisions between mixed outcomes often employs a single model of valuation, and model comparison work is rare. Our aim in this paper is to encourage empirical scientists to design behavioral and neurobiological experiments that can uncover the functional form of value integration in the brain.

In the first section we review the dominant model of value, which assumes an additive integration of costs and benefits. Alternative cost-benefit integration models draw substantially on our understanding of how risk and delay influence valuation. This influence is therefore reviewed briefly in the second section. The third section describes alternative models of cost-benefit integration, all interactive in nature, with the aim of rendering them accessible for the neuroscience community. These three sections concentrate on theoretical models but draw on some pertinent behavioral work. In the final two sections we provide a selective review of relevant neurobiological work from a computational perspective, focusing on those studies that illuminate the underlying valuation mechanisms.

## The Additive Model of Value Integration

A dominant model for cost-benefit decision-making is expected utility theory (Von Neumann and Morgenstern, 1947). Here the subjective value (*V*) of a choice is computed as the sum of the probability (*p*) weighted utility (*U*) of each of its possible outcomes:

(1) $$V=\overset{n}{\underset{k=1}{\sum }}p_{k}\times U\left(m_{k}\right)$$

In this equation *m* signifies the magnitude of rewards and costs associated with the outcomes the choice entails. The utility function in this theory is typically plotted for positive *m*s where it is concave, with diminishing sensitivity for larger *m*s. Costs are represented by negative *U*s. This is an additive model of valuation because the disutilities of costs are summed with the utilities of beneficial outcomes. A positive *V* favors a decision to act, and if there is more than one option under consideration the action with the greatest expected utility is chosen.

**Figure 1:**
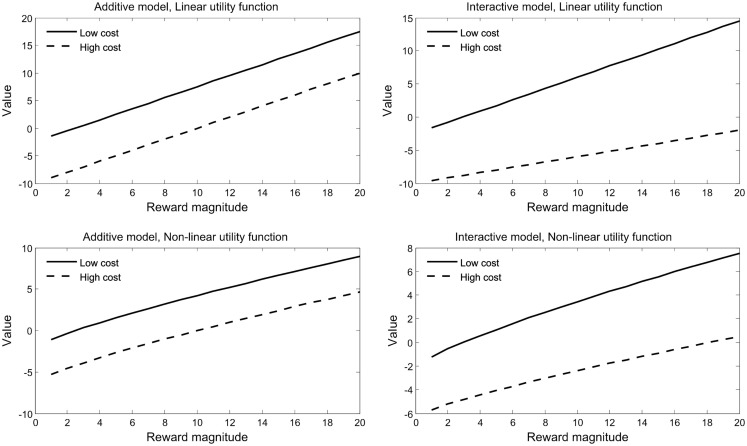
**Additive and. interactive models of value integration**. Value was computed according to the bilinear model with *x*_1_ = 1 and *x*_2_ = 1. Additive models are depicted on the left, with *x*_3_ = 0. Interactive models are depicted on the right, with *x*_3_ = 0.06. Top panels employed a linear utility function for rewards and costs (those that were employed by Talmi et al., 2009); bottom panels employed a power function with exponent 0.8. Additive models with non-linear utility (bottom left) represent the predictions of expected utility theory with *p* = 1. Figure adapted from Park et al. (2011).

The additive model of valuation is dominant, but there are a number of interesting alternatives (see Table 1; Figure 1). Crucially, there are situations in which the additive model may not be valid. Multi-attribute utility theory (Keeney and Raiffa, 1993), for example, allows additive integration only under the assumption of “additive independence” (Thurston, 2006). Consider a lottery where an agent may obtain one of two outcomes with a 50% probability. Both outcomes are a mixture of two attributes, *x* and *y*, each with two values – for example, a large sandwich for $4 or a smaller sandwich for $2. Under additive independence an agent who is indifferent between the two outcomes of Lottery A, [*x*_2_,*y*_1_] and [*x*_1_,*y*_2_] would also be indifferent between the two outcomes of Lottery B where the same attributes and values are recombined [*x*_1_,*y*_1_] and [*x*_2_,*y*_2_]. Clearly, an agent who is indifferent between the possible outcomes of lottery A – the high-reward/high-cost outcome and the low-reward/low-cost outcome – is unlikely to be indifferent between the two outcomes of lottery B where the high-reward/low-cost outcome (a large sandwich for $2) clearly dominates the low-reward/high-cost outcome (a small sandwich for $4). In this scenario reward and cost are not additively independent, suggesting that they should not always be combined according to the additive model.

**Table 1:** **Models of value integration**.

Additive models	Interactive models
Expected utility theory: *V *= *p*_r_ × *U*(*m*_r_) − *p*_c_ × *U*(*m*_c_)	Trade-off model: *V *= *p* × *U*(*m*_r_)/*U*(*m*_c_)
Prospect theory: *V *= *P*(*p*_r_) × *U*(*m*_r_) − *P*(*p*_c_) × *U*(*m*_c_)	Hyperbolic discounting: *V *= *U*(*m*_r_)/[1 + *k* × *U*(*m*_c_)]
Discounted utility theory: *V *= *P*(*p*_r_) × *D*(*d*_r_) × *U*(*m*_r_)− *P*(*p*_c_) × *D*(*d*_c_) × *U*(*m*_c_)	Right-shift of the utility curve: *V *= *P*(*p*) × *U*(*m*_r_ − *m*_c_) Bilinear model: *V *= *x*_1_ × *U*(*m*_r_) − *x*_2_ × *U*(*m*_c_)− *x*_3_ × *U*(*m*_c_) × *U*(*m*_r_)

*The subjective value of a single mixed outcome with probability *p*, one rewarding attribute *m*_r_ and one costly attribute *m*_c_ (coded positively so that greater *m*_c_ represents greater cost), delivered after a time delay *d*. *U*, *P*, and *D* are functions that transform externally given quantities *m*, *p*, and *d* into internal representations. *x*_1_, *x*_2_, and *x*_3_ are constants*.

Before we describe alternative models of cost-benefit integration we discuss in a little more detail how risk and delay are thought to modulate the value of an outcome with either a rewarding or a costly attribute.

## Modulation of Costs and Benefits by Risk and Delay

Consider a patient who must evaluate a treatment option. The improvement in health this treatment brings and the painful procedure it involves must both be weighed against the chance that it is not efficacious and will only yield benefits after a long recovery period. In this section we discuss how risk and delay influence the subjective value of a reward or a cost.

### Modeling risk

Expected utility theory, a prescriptive model for decision-making under risk, fits empirical data less well than the descriptive Prospect theory (Kahneman and Tversky, 1979; Tversky and Kahneman, 1992). In Prospect theory utilities are again computed as a product of two functions, one transforming gains and losses (the utility function) and the other transforming given probabilities (the probability weighing function). The utility function is concave in the gain domain and convex in the loss domain with a steeper slope in the latter, so that the disutility of losses is greater than the utility of gains, allowing prospect theory to account for loss aversion. Prospect theory also proposes a non-fixed reference point for changes in utility, rather than a fixed point representing final wealth states; this feature is important for our discussion in Section “Modeling costs as right-shifts of the utility function”. These transformations allow the theory to account for a number of expected utility violations, such as the reflection and framing effects and biases in the perception of small and large outcome probabilities (Kahneman and Tversky, 1979, 2000; Tversky and Kahneman, 1981). Both expected utility and Prospect theory entail a multiplicative integration of utility with its probability, such that utility is weighted (or discounted) in accordance with its decreasing likelihood of occurrence.

**Figure 2:**
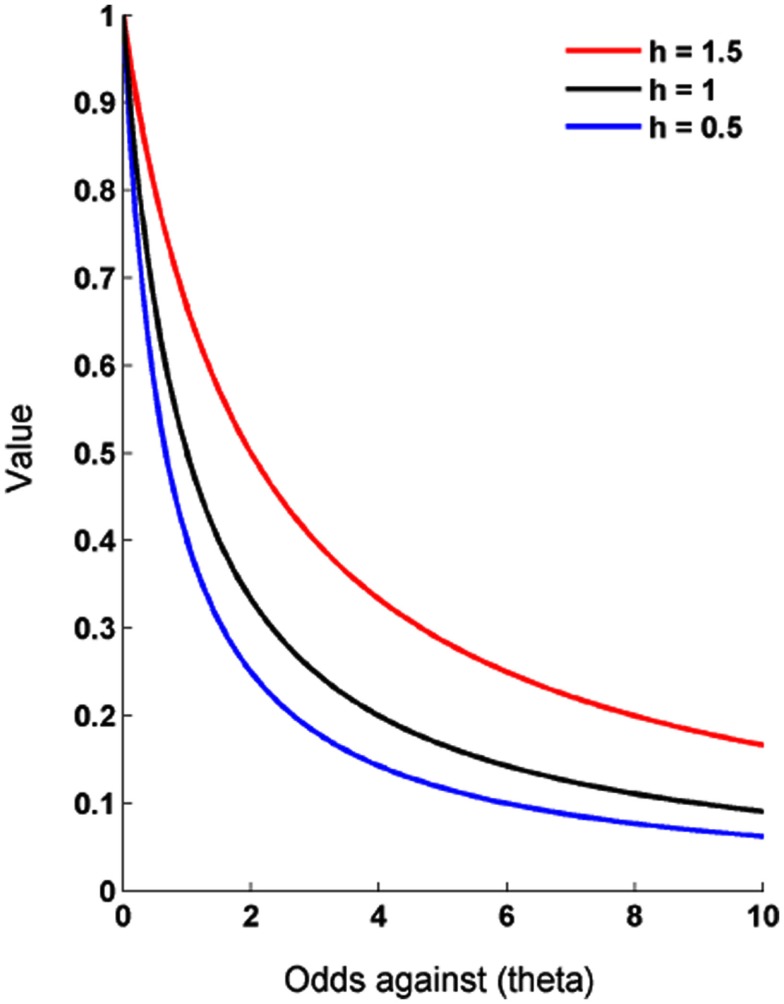
**Hyperbolic discounting of risk**. In this basic hyperbolic model individuals steeply discount reward value with initial decreases in probability of occurrence and only gradually as they get more unlikely. *h* is a risk aversion parameter. In the case of *h* = 1 discounting conforms to EU theory, i.e., an equal decrease in value for every percent decline in probability. *h* < 1 equates to risk aversion and when greater than one to risk seeking. Value here is represented as a proportion of its initial (certain) value or alternatively as the discount factor.

The form of probability discounting proposed by expected utility theory does not account adequately for an array of anomalies in decision-making under uncertainty, but prospect theory can account for most of those. The mathematical form of the probability weighting and utility functions in prospect theory are not formally specified beyond their qualitative predictions, but more precise formulations derived from a body of animal and human probability discounting experiments indicate that probabilistic rewards are discounted in a hyperbolic or quasi-hyperbolic manner as their likelihood diminishes (Green and Myerson, 2004; Green et al., 2004, 2011). These experiments typically employ psychophysical, “adjusting amount” procedures (Rachlin et al., 1991). In a standard procedure, participants are required to choose between a smaller-certain reward and a larger-probabilistic reward. In each trial the amount of the smaller reward is adjusted until the participant is indifferent between the two options. Under the assumption that indifference entails equality of subjective value the subjective value of the risky option can be quantified in terms of the certain (risk-free) option. The probability of the larger reward is then altered such that the probability discount function can be estimated from a number of indifference points across the probability spectrum. Results of these procedures consistently show that hyperbolic functions provide a superior fit to these indifference points, in contrast with the predictions of expected utility theory (Figure 2). To take this into account *p* in Eq. 1 can be replaced by *P*(*p*) where

$$\begin{array}{cc} P\left(p\right)=\frac{1}{1+h\times \Theta } & \text{(1.1)} \end{array}$$

With

$$\begin{array}{cc} \Theta =\frac{1-p}{p} & \text{(1.2)} \end{array}$$

Θ is termed the “odds ratio” and is computed as the probability of non-occurrence divided by the probability of occurrence. An odds ratio of 1 therefore corresponds to outcomes that occur 50% of the time. *h* is a discount rate parameter which determines the rate of probability discounting. If *h* = 1 the individual is risk neutral and values the reward in accordance with EU theory, so *P(p)* = *p*. When *h* < 1 the individual is described as risk averse [*V* < (*p* × *M*)] and when *h* > 1 as risk seeking (Figure 2). *P(p)* can be considered as a discount factor between zero and one by which the reward is discounted in accordance with its “odds against.”

The main feature of a hyperbolic discount function is that the reward loses a gradually smaller proportion of its value per increasing unit in odds against – so it loses a larger proportion of its value when the probability changes from 90 to 80% than when it changes from 60 to 50%. This can explain why a person who chooses a smaller but more certain reward over a larger but more risky option can switch their preferences when the probability of both options is reduced by a constant – similar to the Allais or certainty paradox (Allais, 1953; Kahneman and Tversky, 1979). The smaller (below 1) is *h*, the greater is the steepness of the initial devaluation relative to the later devaluation. Note that this formulation is consistent with the predictions of prospect theory, for example, the overweighting of events with small probabilities and the underweighting events with large probabilities (Figure 2). A number of similar but more complex functions have been proposed that attempt to capture other features of subjective valuation by adding extra parameters (Green and Myerson, 2004; Myerson et al., 2011).

### Modeling delay

While it is difficult to extend EU and prospect theory to account for situations where people choose between rewards or punishments that are available at different points in time (termed intertemporal choice), discounted utility theory models this situation explicitly. The subjective value of a single, certain outcome *m* that is delayed by *d* can be expressed as

(2) V=D(d)×U(m)

With *U*(*m*) representing the instantaneous utility of *m* and *D*(*d*) representing a discount factor ranging from zero to one by which *U*(*m*) is discounted in accordance with its objective delay.

In fact, the probability discounting approach outlined above derives from an older and richer literature on temporal discounting (Loewenstein and Elster, 1992; Frederick et al., 2002; Green and Myerson, 2004), and relates to a debate as to which is the primary discounting mechanism – probability (because delay entails uncertainty) or delay (because the resolution of uncertainty takes time, Prelec and Loewenstein, 1991; Rachlin et al., 1991). In discounted utility theory the utility of a reward is discounted exponentially as a function of its delay, namely with a constant percentage decrease in value per unit time (Samuelson, 1937; Koopmans, 1960).

$$\begin{array}{cc} D\left(d\right)={\text{e}}^{\left(-k*d\right)} & \text{(2.1)} \end{array}$$

*k* is a free parameter which represents the individual’s discount rate. Thus *k* quantifies an individual’s tendency to discount future costs and benefits. An individual with a high *k* value devalues future costs and benefits more steeply than a lower *k* individual, i.e. with a greater percentage decrease in value per unit time. *k* is thought to relate to impulsivity in the same manner as *h* relates to an individual’s risk profile (Ainslie, 1975, 2001) because individuals with a large *k* are more likely to choose the smaller-sooner over larger-later option.

Although people do discount exponentially in some situations (Schweighofer et al., 2006), there is a wealth of empirical evidence against exponential discounting, primarily in the robust finding that the discount rate is not constant but decreases with time. In a simple demonstration (Thaler, 1981) asked subjects to specify the amount of money they would require in 1 month, 1 year or 10 years to make them indifferent between that option and receiving $15 now. Their median responses ($20, $50, $100) implied an average annual discount rate of 19% over a 10 year horizon, 120% over a 1 year horizon and 345% over a 1 month horizon. Similar observations have been made for in non-monetary domains such as health and credit markets (Redelmeier and Heller, 1993; Chapman and Elstein, 1995; Chapman, 1996, 2001; Pender, 1996). A noted manifestation of this feature is that humans and animals are prone to preference reversals when a constant delay is added to both options of an intertemporal choice (Prelec and Loewenstein, 1991; Loewenstein and Elster, 1992). For example, people who prefer $10 today over $11 tomorrow often also prefer $11 in 31 days to $10 in 30 days (Green et al., 1994). As we have seen, the same reversals also characterize choices between certain and probabilistic outcomes.

When mathematical functions are fit to intertemporal choice data (for example indifference points between smaller-sooner and larger-later options) a multitude of studies have demonstrated that hyperbolic or quasi-hyperbolic discount functions provide a superior fit compared to exponential functions in both humans and animals, for delayed monetary, health-related, and other forms of reward, and punishment (reviewed in Rachlin et al., 1991; Ho et al., 1999; Frederick et al., 2002; Green and Myerson, 2004, but see Kable and Glimcher, 2007, for a different model). The standard and most widely used functional form for hyperbolic discounting in the behavioral literature was proposed by Mazur (1987) and based on earlier work by Ainslie and Herrnstein (1981), Ainslie (1975), Herrnstein (1981). According to this work,

$$\begin{array}{cc} D\left(d\right)=\frac{1}{1+k\times d} & \text{(2.2)} \end{array}$$

so that

$$\begin{array}{cc} V=\frac{m}{1+k\times d} & \text{(2.3)} \end{array}$$

If we taker *U*(*m*) to be a better representation of the instantaneous value of *M* (Pine et al., 2009) then

$$\begin{array}{cc} V=\frac{U\left(m\right)}{1+k\times d} & \text{(2.4)} \end{array}$$

As with probability discounting, other functional forms which capture decreasing rates of discounting and the non-linearity of the relationship between objective and subjective delay have also been proposed (Phelps and Pollak, 1968; Loewenstein and Prelec, 1991; Frederick et al., 2002; Green and Myerson, 2004; Myerson et al., 2011).

**Figure 3:**
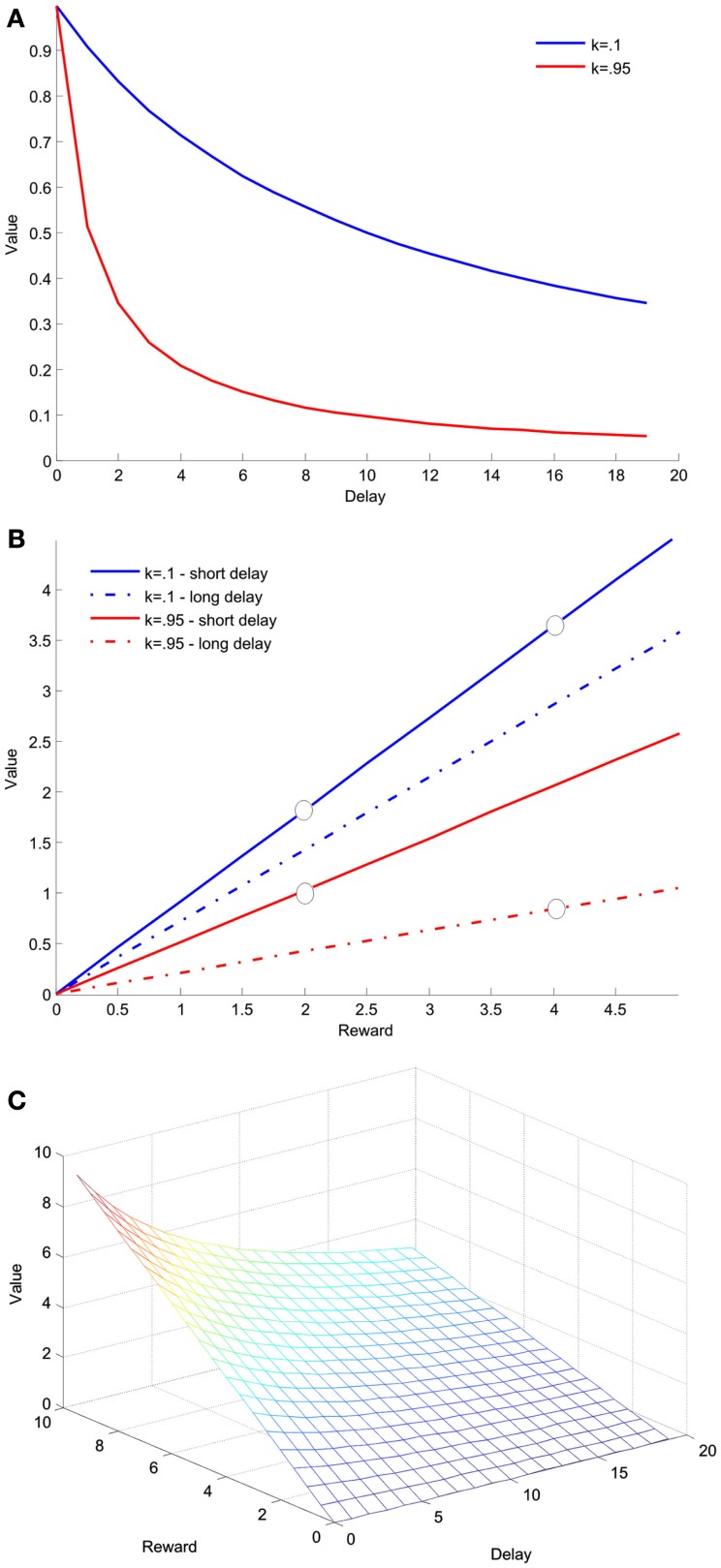
**Hyperbolic discounting of delay**. This function describes theoretical data from an experiment in which two groups of animals are given a choice between two arms of a maze, one of which contains a larger-later reward, four food pellets that necessitate a wait of 15 s, and one which contained a smaller-sooner reward, two food pellets that were available after 3 s. **(A)** Value as a function of delay for a single reward magnitude, two food pellets, computed according to Eq. 2.3. The discount function is depicted for two values of *k* with higher *k* indicating steeper, (more impulsive) discounting. **(B)** Value as a function of reward magnitude for the two different and delays, 3 and 15 s, computed using the same equation. The circles show the two options presented to the two groups of animals. In this example the value of the larger-later reward is greater for the less impulsive group, and the value of the smaller-sooner reward is greater for the more impulsive group. **(C)** Value as a function of both delay and reward magnitude.

Expected utility, prospect theory and discounted utility theory entail an attenuation of reward sensitivity with risk or delay. This means that when reward is risky or delayed, the utility gained by increasing it by a constant reduces, so that *U*(*m *+ 1) − *U*(*m*) is greater than *U(m *+ *1*) × *D*(*d*) − *U*(*m*) × *D(d)* or *U(m *+ *1)* × *P(p) *− *U(m)* × *P(p)*. Figure 3B illustrates this point. By combining these forms of discounting, the subjective value of either costs or benefits can also therefore be represented as a product of utility with two discount factors, one based on probability and the other on delay (Prelec and Loewenstein, 1991; Rachlin and Raineri, 1992; Ho et al., 1999):

$$\begin{array}{cc} V=\overset{n}{\underset{k=1}{\sum }}P\left(p_{k}\right)\times D\left(d_{k}\right)\times U\left(m_{k}\right) & \text{(2.5)} \end{array}$$

### Alternative models for the effects of risk and delay

A key challenge for the models we presented for the integration of reward with probability and delay (Eq. 2.5) concerns the effect of reward magnitude on valuation. The “magnitude effect” refers to a prevalent finding in intertemporal choice that small magnitudes are discounted more steeply than large ones. A person who is indifferent between $60 today and $120 in 1 year is thus more likely to choose $1200 in 1 year to $600 today. The magnitude effect has been documented in numerous studies involving both real and hypothetical rewards (reviewed in Frederick et al., 2002; Green and Myerson, 2004) For instance, Thaler (1981) asked his participants to decide between a given immediate monetary reward and a delayed monetary reward they would receive in a year’s time. Participants were required to declare how much money they would want in a year for them to be indifferent between the immediate and the delayed options. He found that the immediate amounts of $4000, $350, and $60 were discounted by 29, 34, and 39%, respectively. Although the magnitude effect has also been documented in non-monetary reward domains such as medical treatments, drugs, job choices, vacations, and restaurant tips (Raineri and Rachlin, 1993; Chapman and Elstein, 1995; Chapman, 1996; Chapman and Winquist, 1998; Baker et al., 2003; Schoenfelder and Hantula, 2003) it has not been observed in species other than humans, for example in rats and pigeons and primates using food rewards (Richards et al., 1997; Grace, 1999; Green and Myerson, 2004; Freeman et al., 2012, but see Grace et al., 2012). In humans the magnitude effect has not been reliably observed in the loss domain (Estle et al., 2006) and in some (but not all) studies seems to level off when the magnitudes involved are fairly large (Shelley, 1993; Green et al., 1997).

Although less explored, there is evidence that reward magnitude has the opposite effect on probability discounting compared to temporal discounting. The “peanuts effect” describes the finding that larger magnitude rewards are discounted more steeply than smaller rewards, implying that people tend to be less risk averse when they are “playing for peanuts” (Weber and Chapman, 2005; Chapman and Weber, 2006). For example, an individual may prefer a 10% probability of obtaining $100 over a certain $10, but may also prefer a certain $100 to a 10% probability of winning $1000. The reward magnitude effect humans display when they evaluate risky and delay outcomes poses a challenge for the multiplicative *P* × *D* × *U* approach in Eq. 2.5, since it suggests that *D* and *P* themselves depend on *U*. The double dissociation between the effect of reward magnitude on delay and probability exacerbate the challenge because it suggests that magnitude effects cannot simply be explained by a property of the discount function *U*. We briefly review two approaches to this challenge and in Section “Using Neurobiological Evidence to Decide Between Models of Risk and Delay” we discuss how neurobiological data can help decide between them.

Green and Myerson (2004), Myerson et al. (2011) posit that magnitude scales the temporal discount rate parameter *k* in Eq. 2.3 such that *k* decreases with increasing magnitude. By contrast, in probability discounting magnitude scales an exponent of the denominator of the discount function in Eq. 1.1. Because this renders the two discount functions *D* and *P* partially a function of *m*, the subjective value *V* can no longer be thought of as the product of a multiplication of separate utility and discount functions *U*, *D*, and *P*.

Prelec and Loewenstein (1991) offer a general scheme with which to view multi-attribute choice. Their model, an extension of prospect theory, similarly relies on the decomposition of valuation into separate discount and utility functions, and explains magnitude effects in delay discounting in terms of the utility function. They suggest that agents represent or “frame” each attribute of a multi-attribute outcome as a pair of values. The first value is the absolute magnitude of the attribute and the second is what they call the “polarity,” namely, whether it is beneficial or detrimental. For example, $50 in 2 weeks is encoded as (50, 2) with the polarities (+, −). The importance of attributes relative to each other can be altered by certain linear transformations. One such linear transformation is adding a constant to the magnitude of all values of an attribute. The consequence of this transformation is “decreasing absolute sensitivity,” a decrease in the importance of that attribute relative to others. A second transformation involves multiplying all values of an attribute by a constant. The consequence of this transformation is “increasing proportional sensitivity,” an increase in the importance of that attribute. The magnitude effect in intertemporal choice follows from increasing proportional sensitivity because multiplying the monetary attribute increases its importance relative to the delay attribute, leading to the appearance that larger magnitudes are discounted less. These features of multi-attribute framing can explain many of the anomalies common to decision-making under uncertainty and intertemporal choice. Yet because increased proportional sensitivity will always increases the importance of the monetary attribute this effect cannot explain the opposite effects of reward magnitude on delay and probability discounting.

To account for the peanuts effect Prelec and Loewenstein (1991) invoke “interaction effects.” These are emotional processes that can influence cost-benefit decisions by changing the importance of the attributes of an outcome through valuation processes unrelated to utility and discount functions (Berns et al., 2007). Disappointment, one of many interaction effects, accounts for the magnitude effect in probability discounting. The notion here is that anticipation of disappointment – should a losing outcome occur – increases the greater the potential gain, and that increasing disappointment decreases the importance of the money relative to the probability attribute in a manner that accounts for preference reversals (Prelec and Loewenstein, 1991; Weber and Chapman, 2005; Chapman and Weber, 2006). Disappointment does not enter into delay discounting since there are no probabilistic outcomes. Interaction effects are useful, however, when we consider other interesting phenomena in intertemporal choice. The interaction effects of anticipation and dread are invoked to explain why in some cases people prefer to speed up punishments to “get them over with,” and savor rewards by delaying them, phenomena which are incompatible with standard discounted utility theory (Loewenstein, 1987; Berns et al., 2006).

In summary, the discounted utility theory notion of a decision value-based on the multiplication of separate utility and discount functions has been challenged in light of opposing magnitude effects. In one view discount functions accept magnitude as an argument, with no requirement for a separate utility function. Although two separate mechanisms are required to account for opposing magnitude effects this is perhaps a more parsimonious account, but it does not explain a host of other influences on valuation that are captured by interaction effects. In another view additional mechanisms are invoked with magnitude solely acting on the utility function, and delay and risk are treated within separate weighting functions.

## Interactive Models of Value Integration

While the functional form of decision-making that involves risk and delay costs is well described, and a rich empirical literature delineates the neurobiology of effort-based decision-making (Salamone et al., 2007; Floresco et al., 2008a; Kurniawan et al., 2011), less research has been devoted to uncovering the functional form of valuation when effort and other costs are mixed with reward. In this section we review non-additive models for decision values when outcomes include costs. Because the empirical evidence for these models is more limited than that of the additive model we describe them within the context of experiments that corroborate them. Our aim is to expose these models for scrutiny by the neuroscience community and encourage further model comparison work to decide between them. Table 1 lists all the valuation models discussed in this paper.

A strong alternative to the additive model is the trade-off model (Simonson, 1989), where decision values are expressed as the ratio of costs and benefits. The subjective value of a single mixed outcome with probability *p*, one rewarding attribute *m*_r_ and one costly attribute *m_C_*, could be expressed as:

(3) $$V=p\times \frac{U\left(m_{r}\right)}{U\left(m_{C}\right)}$$

Let us take two examples to illustrate how this model has been employed. Soman (2004) used the trade-off model to account for the results of an experiment where participants made hypothetical decisions between differently priced products that required more and less effort, such as an expensive desk that was already assembled and a cheaper desk that required customer assembly. Soman did not utilize model comparison but the trade-off model fitted his data well and accounted for the influence of delay on choosing between these mixed outcomes. Another example comes from work on foraging, where both the additive and the trade-off models are prevalent (Stephens and Krebs, 1986). Bautista et al. (2001) modeled cost-benefit decisions of starlings deciding how to forage for food: to walk (low-reward/low effort) or fly (high-reward/high effort). In that set-up rewards consisted of the energy gain from the food, and costs consisted of the energy loss associated with the chosen travel method. The authors compared the additive model and the trade-off models, which they termed net rate and efficiency, respectively, and found that the additive model accounted best for the starlings’ decision.

One key difference between the additive and trade-off models is how they treat situations that do not offer any reward but impose a cost. The additive model allows subjective values to become negative in these situations, while the trade-off model does not. The importance of this feature depends on the situation. While non-rewarding decisions – between two costs, or a cost and the *status quo* – are rare in economic studies, they are common outside the laboratory. Berns et al. (2008) used an example of a patient deciding between risky treatment options, where the best outcome is maintaining current levels of health, and contended that it is difficult to explore such decisions in typical financial decision-making experiments because people are unlikely to take part in a study where they might end up losing out financially. The difficulty with financial costs is partially ameliorated in experiments that require participants to exert effort or suffer experimentally induced pain, although here too there is an implicit, unmodelled reward that draws participants to take part in the experiment in the first place. Clearly, though, according to the trade-off model the patient’s decision or its laboratory equivalents, do not have a negative decision utility. The next models of valuation that we review differ in their approach to decisions between costs. The first does not model negative utilities in the absence of reward, the second predicts negative utilities, and the third allows for either zero or negative utilities. Therefore, in decisions between “bad” and “worse” an empirical demonstration of negative decision utilities will constrain model selection.

Another key difference between the additive and the trade-off models is whether reward sensitivity changes with costs. This is an important and under-appreciated difference between models. The additive model predicts that costs do not alter reward sensitivity, while the trade-off model predicts that they do. The models we review below differ in this respect too. The first predicts decreased reward sensitivity with cost, the second predicts increased sensitivity, and the third allows changes in either direction. Measuring changes in reward sensitivity is therefore another assay for model selection. Taken together, three aspects of the decision – whether it adheres to the additive independence assumption, the presence or absence of negative decision utilities, and whether reward sensitivity changes with cost – distinguishes integration across costs and benefit from valuation of all-rewarding multi-attribute outcomes.

### Hyperbolic discounting of rewards associated with costs

Brañas-Garza et al. (2012) report a result that illuminates the social importance of understanding decision values of mixed outcomes in the field of health. When people consider a painful medical procedure their decision values should integrate over the pain costs of the procedure as well as the value of consequent future health benefits. How steeply one discounts the future will therefore impinge on the integrated value of the procedure. They found that the more impatient participants, those that discounted the future more steeply in an intertemporal choice task, reported more frequently that they experience a negative feeling as soon as they decide to undergo the procedure. This feeling may derive from the disutility of the decision value, and bias these participants against some health-promoting behaviors such as necessary painful medical procedures.

The success of the hyperbolic discount functions in accounting for the effect of delay and risk costs on choice makes it rather tempting to consider whether other costs devalue reward hyperbolically. The subjective value of a single, certain, mixed outcome with one rewarding attribute *m*_r_ and one costly attribute *m*_c_, could be expressed as:

(4) $$V=\frac{U\left(m_{r}\right)}{1+k\times U\left(m_{C}\right)}$$

Prevost et al. (2010) used hyperbolic discounting (Eq. 2.4) to model how participants decided between cost-benefit mixtures. Participants first viewed fuzzy erotic images and then decided between two options: either viewing a clear image of the same content for a low-cost, or viewing it for a longer duration for a higher cost. The low-cost involved a short wait or exerting minimal physical effort, while the high-cost involved waiting longer or exerting more effort. The hyperbolic discount function described choices equally well for both delay and effort cost, and fared better in doing so than the exponential discount function.

We have seen that hyperbolic models are not ideal for situations that require a cost without providing a reward, because they do not allow negative decision utility when only costs are on offer. However, all trials in the paradigm used by Prevost et al. (2010) included at least a small reward, possibly contributing to the fit of this model for their data.

Clearly, if effort also modulates *U*(*m_R_*) hyperbolically, reward sensitivity will decrease under effort. But a hyperbolic interaction of effort with reward may not be detected in studies that only consider a linear form of interaction. Kurniawan et al. (2010), for example, obtained a different result from Prevost et al. (2010) in a task that similarly required participants to choose between a low-reward, low effort option and a large reward, large-effort option. Both effort and reward had the expected effect on decisions, and a similar effect on ratings of choice likability, but the interaction between reward and effort was not significant for either measurement. The null interaction effect appears to go against interactive models, but as Kurniawan and colleagues used the general linear model in their analysis it is possible that they could not detect a non-linear interaction between effort and reward.

Prelec and Loewenstein (1991) speculated that their rules for transformation of attribute weighting should apply to all multi-attribute choices. Consequently, if effort and pain discount reward hyperbolically it would be natural to predict that effort and pain discounting will resemble risk and delay discounting, and generate preference reversals when a constant is added to both options. In decisions between outcomes that mix reward and pain, for example, adding a constant amount of pain to both options should shift preference in favor of the high-reward high-pain option. This is an example of how consideration of underlying models yields useful hypotheses for experimentation.

### Modeling costs as right-shifts of the utility function

In prospect theory, choice options are coded as gains and losses relative to the “status quo,” a neutral point of reference that is assigned a value of zero on the reward magnitude axis and is where the utility function crosses that axis (Kahneman and Tversky, 1979). Utility is zero when people do not expend any effort, and therefore do not expect to be rewarded. Kivetz (2003) argued that effort requirements create an expectation for reward, which should be modeled as a right shift of the reference point. For example, when Thea is asked to mow the lawn she forms an expectation for reward. Agreeing to do this chore has a negative decision utility and is experienced as a loss relative to her revised reference point. If she is promised $5 for completing this chore and considers this sum fair this reward merely brings her decision value back to zero.

Prospect theory can be extended to account for effort costs by shifting utility function to the right under effort (Kivetz, 2003). People who expend effort *U*(*m_c0_*) expect a fair reward, *U*(*m_r0_*) in return. According to this formulation people should be indifferent between no reward/no effort and *U*(*m_ro_*)/*U*(*m_c0_*). Kivetz (2003) was interested in frequency programs such as frequent-flyer miles, a marketing tool that requires customers to invest effort for future rewards. He showed that replacing *U*(*m*) in Eq. 1 by *U*(*m*_r_–*m*_c_) provided an adequate account for customers’ choices and for the influence of risk on their decisions. The subjective value of a single mixed outcome with probability *p*, one rewarding attribute *m*_r_ and one costly attribute *m*_c_, could be expressed as:

(5) $$V=P\left(p\right)\times U\left(m_{r}-m_{C}\right)$$

Because of the concavity of *U*, right-shifting it under effort means that *U* now increases more steeply with *m_R_*. Consequently, this model implies that effort increases reward sensitivity. For example, Thea may be just a little more delighted with a gift of $10 than $5, but after she is asked to mow the lawn her increased happiness with $10 relative to $5 is greater.

Kivetz (2003) argued that his model can be extended to all costs that people perceive as having an inherent disutility, and mentions delay and pain costs. Beyond the conceptual problem of considering the passage of time as inherently negative we would argue that the empirical evidence base for decreased reward sensitivity with delay (Figure 3) means that Kivetz’ model, which predicts increased sensitivity under cost, is unlikely to account well for intertemporal choice.

### A bilinear model for cost-benefit analysis

Phillips et al. (2007) based their proposed valuation model on their review of animal research concerning the role of dopamine in cost-benefit analysis. Their model was intended to be applicable for delay, effort, risk, and other aversive outcomes. They did not provide the functional form of the value function, perhaps because there is limited evidence for two of the central components of their model, namely, exactly how dopamine levels and the indifference functions vary with reward magnitude. Noting these reservations, and making some assumptions of our own, we derived their value function (Appendix). The function we derived in this way is somewhat unwieldy. However, for small rewards, within the linear portion of the utility function, their model can be expressed more simply as:

(6) $$V=x_{1}\times U\left(m_{r}\right)+x_{2}\times U\left(m_{C}\right)+x_{3}\times U\left(m_{r}\right)\times U\left(m_{C}\right)$$

With positive constants *x*_1_ and *x*_2_, and a constant *x*_3_ that can be either positive or negative. Thus, although the value function proposed by Phillips et al. (2007) may appear to model value additively (Botvinick et al., 2009), a closer look shows that it includes an interaction between reward and cost, albeit of a different form than that in Eqs 3–5. Figure 1 depicts this model and compares it to the additive model both for a linear and for a non-linear utility function.

Two aspect of the bilinear model are important for our discussion. First, in contrast with the other models discussed here this model allows reward sensitivity to either increase or decrease when outcomes include costs. The direction of change depends crucially on the functional forms of the reward utility function and the indifference function (see Appendix). Second, in contrast to the models in Eqs 3 and 4 this model allows utility to become negative when the choice options do not offer any reward.

Although Phillips et al. (2007) offered a very specific functional form to describe the interaction of rewards, costs, and value, they did not describe direct empirical evidence for that particular form. Two studies that examined decisions involving pain costs observed that the bilinear interaction model fitted their data well. In the first study (Talmi et al., 2009) participants chose between two options, one that maximized and one that minimized the chances for the delivery of a mixed outcome. That outcome included a monetary gain or a loss as well as an electrical stimulation of the skin that could be either painful or mild. Participants experienced the pain a few seconds after they made their choice, at which time they were also informed that the promised amount of money was added to their account. When Talmi and colleagues compared the additive model with the bilinear interaction model they found that the addition of an interactive term significantly improved the model fit, with the interaction parameter *x*_3_ suggesting that physical pain attenuated the sensitivity of participants to monetary reward. This conclusion was corroborated by another study (Park et al., 2011) where participants were asked to accept or reject mixed outcomes that involved varying monetary reward and one of five pain magnitudes. The authors replicated Talmi et al.’s (2009) finding that the bilinear model accounted for, behavioral choice better than the additive model. Notably, participants in both studies likely experienced disutility in some of the experimental trials, because Talmi et al. paired pain with either gains, losses, or zero rewards, and Park et al. used a very low amount of 1 cent in some of the trials, paired with both low and high levels of pain. This aspect of their paradigm may explain the importance of the parameter *x*_2_ in their data.

While Talmi et al. argued that because the monetary rewards were very small, under $0.60, the utility of reward and the disutility of pain should be modeled linearly (Rabin and Thaler, 2001), Park et al. (2011) tested this hypothesis formally. Although they also employed small monetary rewards, up to €0.99, they found that modeling reward utility using a power function explained their data better than when a linear function was used. When this change was implemented, behavioral choice data no longer favored the bilinear over the additive model. Their fMRI results, however, fit the bilinear model better than the additive model regardless of whether reward utility was modeled with a linear or a power function. Figure 1 (adapted from their Figure 2) compares four models: two interactive models, computed either with a linear or a non-linear utility function, and their additive model counterparts, which are identical but omit the interaction term. In fact, a detailed analysis of the bilinear model suggests that the importance of the interaction term depends on the relationship between the utility functions for rewards and costs, and is likely not to be important when they are identical. In order to decide, for a particular situation, whether an interaction term is present it is sufficient to determine the form of three functions: the utility function of rewards without costs; the utility function of costs without rewards; and the indifference function – the relationship between rewards and costs. In future work we plan to investigate the conditions for an interaction in more detail.

In summary, while the additive model is dominant in the economic literature, there are several alternative models that feature an interaction between costs and benefits such that costs alter sensitivity to reward. According to four of these models, described in this section, costs modulate the subjective utility of mixed outcomes. Each of these models proposes a different functional form to describe this interaction. Further empirical work can help determine which model best captures the decision values of mixed outcomes in animals and humans. In Section “Using Neurobiological Evidence to Decide between Additive and Interactive Valuation Models” we explore how neurobiological data can assist in this endeavor, but first we explore how such data can shed light on the modulation of costs and benefits by risk and delay.

## Using Neurobiological Evidence to Decide between Models of Risk and Delay

A large amount of empirical work in animals has been dedicated to uncovering the neural structures that mediate decision-making when mixed outcomes involve both costs and benefits. There is strong evidence in human neuroimaging for an abstract representation of subjective utility in the ventromedial prefrontal cortex (vmPFC) across many different kinds of commodities (Padoa-Schioppa, 2011; Levy and Glimcher, 2012), but controversy on where this abstract representation is expressed in animals (Padoa-Schioppa, 2011; Roesch and Bryden, 2011). The regions involved in the effects of delay, risk, and effort on decision-making have been described, with more limited investigations of other costs (Phillips et al., 2007; Salamone et al., 2007; Floresco et al., 2008a; Roesch and Bryden, 2011). Much of this literature, however, does not speak directly to the issues we focus on here, the functional form of integrative valuation of mixed outcomes. In this section we examine how empirical neurobiological data could help decide between the models for the modulation of costs or benefits, separately, by risk and delay. In the final section we discuss data relevant for models of cost-benefit integration.

### Evidence for separate representations of *D* and *U*

Animal and human neuroimaging studies have identified a relatively large set of regions that are involved in intertemporal decision-making (for reviews in animal studies see Cardinal et al., 2004; Winstanley et al., 2006; Floresco et al., 2008a,b; and in humans Tanaka et al., 2004, 2007; McClure et al., 2004, 2007; Kable and Glimcher, 2007; Gregorios-Pippas et al., 2009; Luhmann et al., 2008; Ballard and Knutson, 2009; Wittmann et al., 2007; Prevost et al., 2010; Hariri et al., 2006; Pine et al., 2009, 2010. They include ventromedial and medial prefrontal cortex, dorsal, and ventral striatum (VS), posterior cingulate cortex, and insula, as well as dorsolateral PFC, amygdala, and lateral OFC. There is no agreement on the particular contribution of each region to intertemporal choice and value construction. Recent literature has started to address regional specificity by correlating behaviorally derived model parameters with BOLD responses or single cell electrophysiological recordings. We will demonstrate how this approach has led to an increasingly sophisticated view of functional specificity and model implementation in the brain in animal electrophysiological recording and human neuroimaging studies.

McClure et al. (2004) performed the first neuroimaging study of intertemporal choice to provide a neurobiological account of temporal discounting and preference reversals. The disproportionate valuation of rewards available in the immediate future, and other evidence, led them to postulate the differential activation of distinguishable neural systems – specifically, that impatience is driven by the limbic system which responds to immediate rewards and is less sensitive to the value of future rewards, whereas patience is mediated by the lateral PFC which is able to evaluate trade-offs between more abstract rewards, including those in the more distant future. This proposed struggle between an affective and a deliberative decision-making system was based theoretically on a quasi-hyperbolic time discounting function which splices together two different discounting functions, one exponential and another which distinguishes sharply between present and future rewards, modeled by a parameter termed beta. Beta represents the special value placed on immediate rewards relative to those received at any other time. The hypothesis then was that activity in lateral PFC areas should correspond with the rational, deliberative processes, and limbic activity should represent the beta parameter. To test this hypothesis, they scanned the brains of subjects as they made a series of different hypothetical intertemporal choices. Critically, they split the trials into two types – those where both rewards were delayed in the future, and those where the small reward could be received immediately following the experiment.

When they compared these two conditions in their analysis they found that whereas lateral PFC (dorsal and ventral) and intraparietal regions were similarly active across all trial types, limbic structures including the VS (NAc), mPFC, posterior cingulate, and medial OFC (regions they defined as beta regions) were preferentially activated in response to choices where there was an option for immediate reward. Furthermore, when they analyzed all the choices where there was an immediate component, they could predict the choice outcome – a greater activation of limbic areas led to choice of the immediate small reward, whereas choice of the delayed reward followed a greater activation of the lateral PFC areas relative to the limbic ones. However, the hypothesis that the limbic system mediates impulsivity by its preference for immediate rewards is difficult to reconcile with animal work indicating that the integrity of the NAc and OFC is crucial for self-control and the ability to choose delayed rewards (Cardinal et al., 2004). In McClure et al.’s (2004) account, NAc or OFC lesions should, in theory, promote delayed choice as long as the DLPFC is left intact.

Kable and Glimcher (2007) have argued that McClure et al.’s (2004) study provided insufficient evidence for a dual valuation system since they did not demonstrate activity corresponding to different discount rates in the limbic and lateral PFC regions, and critically, that the discount rate in the beta regions was greater than the observed behavioral discount rate of the subjects. Without such evidence the results of their analysis could simply be explained by proposing that limbic regions track the subjective value of rewards at all delays and this preferential activity simply reflects the fact that sooner rewards are more valuable than later rewards. Thus, to explicitly determine the neural correlates of subjective value in intertemporal choice Kable and Glimcher employed a model-based approach in their analyses. They scanned participants while they were deciding between a constant smaller-sooner option and a variable larger-later option. The crux of their analysis was regressing the BOLD response against the hyperbolically discounted values of the larger-later option, derived from the choices each subject made, by estimating individuals’ discount rate parameter (*k*) according to Mazur’s (1987) hyperbolic discounting function (Eq. 2.3). These regressors identified a network of three regions which correlated with the subjective discounted value of the delayed reward – the VS, medial prefrontal cortex, and posterior cingulate cortex. This network did not exclusively value immediate rewards, as hypothesized by McClure et al. (2004) but tracked the subjective value of rewards at all delays, leading Kable and Glimcher to conclude that there is a single valuation system for delayed rewards.

Kable and Glimcher (2007) along with subsequent studies (Peters and Buchel, 2010; Prevost et al., 2010) successfully established a neural correlate of the subjective value of delayed rewards under the assumption of a single valuation process, but did not attempt to tease apart the putative subcomponents of this process. Therefore, their data cannot distinguish between a single valuation system in Green and Myerson’s (2004) model and the multiplicative model in Eq. 2.5. To examine the architecture of valuation in more detail Pine et al. (2009) scanned participants while they were deciding between serially presented options that differed in both monetary amount and delay. Here the BOLD responses during the presentation of each option were modeled with the three key subcomponents *U*, *D*, and *V*, derived from subjects’ choices according to Eq. 2.5. To ensure that no brain activity could be misattributed to a particular regressor by virtue of correlation with another, these regressors were orthogonalized. Pine et al. found that *U* correlated with activity in ventral tegmental area (VTA), striatum, and anterior cingulate cortex (ACC); *D* with VTA, striatum, insula, posterior cingulate cortex, inferior frontal gyrus, and vmPFC; and *V* with dorsal striatum and subgenual ACC/vmPFC. Interestingly, there was one anatomical region in the dorsal striatum where all three independent correlations overlapped, that is this area correlated independently with the discount factor, utility, and subjective value.

These results demonstrated that the brain evaluates delayed rewards in an integrative fashion. They suggest that the determinants of value are estimated separately, both with a system which relates instantaneous, undiscounted subjective value to the magnitude dimension, and with a system which calculates a discount factor to evaluate the subjective value of rewards based on their delay. Finally, a further set of regions encodes the multiplicatively integrated value of these subcomponents, which is then used to guide decisions. It was demonstrated that the dorsal striatum is the site where information from the individual value systems is integrated, because only this region represented all three subcomponents. Pine et al. (2009) thus replicated Kable and Glimcher’s (2007) findings of subjective value coding in the striatum and medial PFC, but extended them to demonstrate an expanded network of regions involved in discrete aspects of the multiplicative valuation process. To illustrate the difference consider the possibility that the activity in the posterior cingulate cortex reported by Kable and Glimcher expresses the discount factor rather than overall subjective value (i.e. implicating this region solely in discounting) – a possibility supported by Pine et al.’s findings that this region only correlated with *D*, and not *V*. These results are thus consistent with the separation of *D* and *U* (Eq. 2), and support the Prelec and Loewenstein (1991) framework over the notion of a single valuation process.

Working on a similar premise, over a number of experiments, Roesch et al. (2007), Roesch and Bryden (2011) recorded from single units in rats as they made intertemporal choices. They manipulated magnitude and delay over different blocks in order to understand how these two determinants of value are represented and integrated in various brain regions. They found that activity in the majority of the OFC neurons they recorded declined as the delay to the reward increased, implicating this region in the temporal discounting of rewards. This activity also correlated with a decreased tendency for rats to choose the larger-later option trials. Interestingly, Roesch et al. (2007) argue that the OFC does not represent *V* and that the OFC is not a site of “common value currency” because neurons that encoded delay did not also encode reward magnitude (a requirement if they encoded discounted value). These results conflict with other electrophysiological findings, which observed delay-discounted values of reward in single neurons in pigeon OFC analog (Kalenscher et al., 2005) and in primate OFC (Roesch and Olson, 2005). Additionally, they seem to contradict the finding that OFC lesions in rats influence both temporal discount rates and sensitivity to reward (*D* and *U*, Kheramin et al., 2002, 2003, 2004). Taken together, it is not yet clear whether the animal medial PFC represents *U*, *D*, and *V* separately or in an integrated fashion.

Of all the single neurons recorded in rodents by Roesch and Bryden (2011) only dopamine neurons in the midbrain, especially in VTA, appeared to integrate magnitude and delay in that they encoded to both variables and their responses to the two variables were highly correlated. As a population, neurons in VS also encoded both delay and magnitude (see also forced choice voltametry data in Day et al., 2011), and some neurons did responded to both variables in a correlated manner, but overall the correlation between the response of single cells to these two variables was low. Kobayashi and Schultz (2008) demonstrated more specifically that the activity of midbrain dopamine neurons in primates tracks the discounted value of rewards in accordance with a hyperbolic discount function. Neural firing in response to Pavlovian conditioned stimuli that predicted rewards of differing delays decreased with longer delays, at a rate similar to the discount rate measured when the same animal performed a separate choice task, and followed a pattern akin to a hyperbolic decline. These neurons were also responsive to the magnitude of the predicted reward. The site of integration Pine et al. (2009) localized in the striatum may therefore reflect the output of midbrain dopamine neurons in the rodent electrophysiological recordings (Logothetis et al., 2001). Alternatively, Pine et al.’s findings could be related to coding of temporally discounted rewards in the primate dorsal striatum reported by Cai et al. (2011).

In summary, animal and human work converge on a network comprising VTA, striatum, and medial PFC which is involved in computing and representing subjective value, but the exact role of each of these regions in constructing value remains debated. Evidence from animal single unit recordings corroborates the hierarchical model of separate encoding of *D* and *U* with an integration of the two to inform subjective value, but there is a controversy as to how downstream one has to record to locate the site of this integration. In human fMRI studies, by contrast, correlates of subjective value derived from hyperbolic discount functions are fairly consistent, but only one paper so far has investigated each component of the multiplicative model individually to delineate regions implementing separate valuation functions for delay and magnitude and their integration.

### Evidence for separate representations of *P* and *U*

As with temporal discounting, a burgeoning human neuroimaging literature has shed a great deal of light on the neurobiological mechanisms of valuation and decision-making under uncertainty. Early studies examined BOLD responses to anticipation versus outcome of probabilistic rewards of varying magnitude (Breiter et al., 2001; Knutson et al., 2001a,b). Subsequent studies have identified the neural regions performing computations relating to the subjective value of risky prospects based on their expected values – correlates of which have been observed in the striatum and OFC (Preuschoff et al., 2006; Rolls et al., 2007; Tobler et al., 2007). The utility and probability functions which are multiplied to calculate subjective value are typically assumed to be linear and their neural correlates have also been examined individually (Knutson et al., 2001a examined magnitude and Abler et al., 2006 examined probability). In addition, more sophisticated Prospect theory-like utility and probability functions have been associated with BOLD signal in the striatum and vmPFC when participants decide between gambles (Tom et al., 2007; Hsu et al., 2009). Another school of thought proposes that in addition to expected values/utilities, the “riskiness” of probabilistic rewards is encoded by the brain and has some role in valuation. Such properties can be modeled by statistical moments such as their mathematical variance and skewness. Correlates of the former have been found in the lateral OFC, insula, and striatum (Dreher et al., 2006; Preuschoff et al., 2006, 2008; Rolls et al., 2007; Tobler et al., 2007; for detailed reviews see Rangel et al., 2008; Schultz et al., 2008).

**Figure 4:**
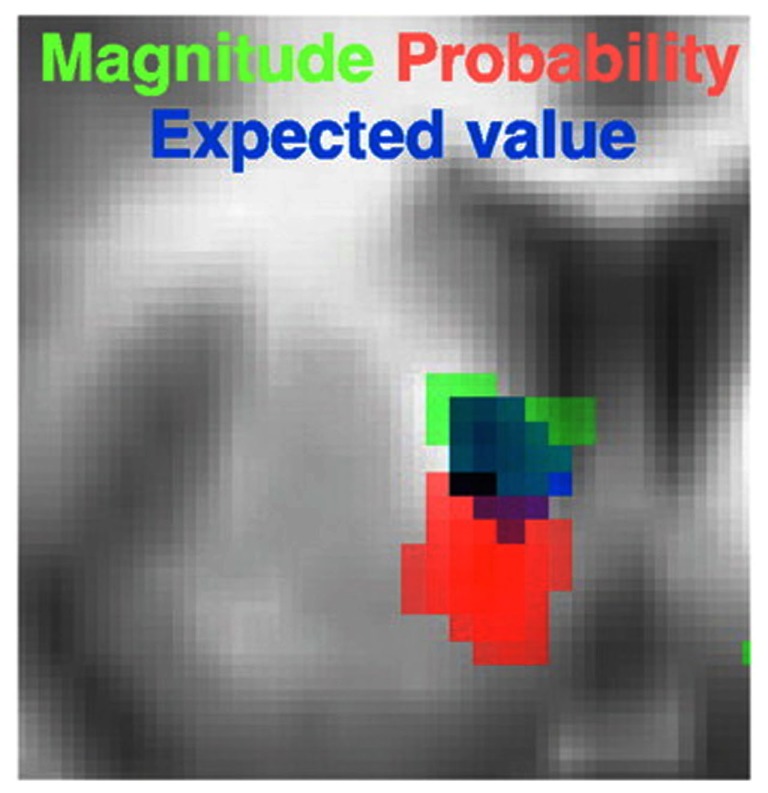
**Separate and partially overlapping striatal regions encoding unique valuation components of probabilistic rewards**. Activity in dorsal striatum positively correlated with *U* (in this case magnitude), and in ventral striatum with probability. Their product, that is expected utility, positively correlated with a third, and overlapping striatal region. Figure adapted from Tobler et al. (2007).

Surprisingly however, to our knowledge only one study has attempted to tease apart the three components of the model in Eq. 1 within the same task. Tobler et al. (2007) presented their participants with stimuli predictive of outcomes which varied in their probability and magnitude. The value of each cue was computed as the product of the given, objective probability of the associated outcomes and their utility, which was modeled simply as a linear function of magnitude. They found that both the magnitude and probability of the predicted outcome correlated positively with separate regions in the striatum (dorsal and ventral respectively; Figure 4). In contrast, the medial PFC was only responsive to the probability of each reward. When correlating the expected value of each option predicted by the cue, Tobler et al. (2007) observed a third and separate region in the striatum. Critically, this region also overlapped with the individual probability and magnitude sensitive regions (Figure 4), strongly suggesting the striatum a site for the integration of probability and utility. To make the case more convincing Tobler et al. (2007) also showed that the striatal BOLD response to a particular expected value was the same whether the value was a product of a large magnitude and low probability (e.g., 200 points with a 10% probability) or vice versa (e.g., 20 points with a 100% probability). In a prior study (Fiorillo et al., 2003) the same group showed that VTA neurons exhibited similar characteristics, correlating positively with probability, magnitude, and their multiplicative integration. Tobler et al.’s results are analogous to those of Pine et al. in the intertemporal sphere, in that both studies revealed that each component of the integrative model in Eq. 2 was represented separately in the brain. The findings of Tobler, Fiorillo, Pine, and their colleagues therefore support the multiplicative integration of separable value components rather than a single valuation function, as in the model advocated by Green and Myerson (2004).

Berns and Bell (2012) recently utilized a task where information about the probability and magnitude of rewards were presented sequentially. Although they did not look for correlates of expected value they did assess probability and magnitude independently and showed that whereas magnitude correlated with the ventral striatum BOLD responses, probability correlated with dorsal striatum activity. In this instance however the two regions did not overlap. This led the authors to conclude that although magnitude and probability are processed by distinct neural systems, their integration is not achieved by any of the models discussed above. We note, however, that none of the studies discussed in this section fully embraced the model in Eq. 1 in that they all used objective magnitudes and probabilities instead of their transformation by the utility and the probability weighting functions. This is more of a concern for null results which could be more easily explained by parameter misspecification. Therefore, before accepting Berns and Bell’s conclusion it would be necessary to check whether an overlap could be found when BOLD signal is regressed against participant-specific, behaviorally derived utilities, and subjective probabilities.

### Evidence for separate representations of *D* and *P*

Both Tobler et al. (2007) and Pine et al. (2009) found that magnitude correlated predominantly with the striatum, whereas the modulators of magnitude – delay and risk – were expressed in the striatum and vmPFC among other regions. In both studies the striatum was considered the critical site of value integration. This begs the question of whether probability and delay share neural mechanisms, and even whether all modulators of reward are integrated in the striatum.

In support of the dissociation between *D* and *P*, Simon et al. (2009) observed poor correlation between the two across individuals. They allowed rats to choose between a safe small reward and a larger reward that was associated with varied probability of an electric shock. As expected, increasing magnitude and probability of shock biased rats to prefer the safe reward, and individual animals showed a stable pattern of responding across training sessions. The same rats also took part in probability discounting and delay discounting tasks that entailed choice between rewards without risk of shock. The sensitivity of individual animals to the risk of shock – namely, their tendency to opt for the safe reward in the main task – was correlated with sensitivity to risk in the probability discounting task but not with sensitivity to delay in the delay discounting task.

Relatively few fMRI studies have studied probabilistic and intertemporal choices in the same task (Luhmann et al., 2008; Ballard and Knutson, 2009; Peters and Buchel, 2009). Of these, only Peters and Buchel employed a model-based approach. They scanned participants while they were deciding between an immediate-small and larger-later rewards, and separately, between a certain-small and larger-probabilistic rewards. Decisions were modeled using hyperbolic discount functions to infer participant-specific parameters for *k* and *h*, which were subsequently used to calculate the subjective value of the dynamic (larger-later, larger-risky) options. Utility was modeled as a linear function of reward magnitude. To demonstrate that subjective value here was equivalent across choice types participants also performed a separate behavioral experiment where they decided between delayed and risky rewards. Indeed, Peters and Buchel found that their participants were indifferent between delayed and probabilistic rewards which had the same subjective value as calculated from the separate tasks, indicating the rewards had comparable intrinsic values. Analyses of the BOLD response revealed both overlapping and diverging activations. Whereas overlapping regions in the striatum and OFC were correlated with subjective value in both cases, other regions correlated with the subjective value of either risk- or delay-discounted reward. The authors concluded that the striatum and OFC are domain-general valuation regions which integrate results from domain specific subjective valuation systems into a common “neural currency” of value – that is a metric of value which can be used to compare the utilities of various multi-attribute options.

Though this is certainly a feasible interpretation, it is somewhat unintuitive to assume an integration of different subjective values (*V*s) rather than an integration of different sub-components of a common subjective value (such as *U, D*, and *P*). Indeed, the analysis performed by Peters and Buchel (2009) does not eliminate the possibility that some of the regions identified as correlating with *V* could in fact have a more specific role in the representation of one of the subcomponents of subjective value^1^. Had they have compared brain activity which could only be explained by utility, the discount factors *D* and *P* (rather than inverse delay and probability), and subjective value we would have a clearer picture of the relationship between each domain specific discount system, if they are common or separable, and where and they are integrated with utility to calculate subjective value.

A critical experiment to elucidate integration across reward and its modulators, delay and probability, would be to examine the BOLD correlates when participants decide between options with all three attributes, i.e., delayed probabilistic rewards. Modeling such choices will enable a neurobiological evaluation of the multiplicative *V* = *U* × *D* × *P* approach outlined in Eq. 2.5.

## Using Neurobiological Evidence to Decide between Additive and Interactive Valuation Models

The claim that animals and humans represent a mixed outcome with a single value, and that this value is intimately tied to subsequent choice, is dominant in neuroeconomics and fundamental for the models we discussed here (although it is not without its critics: Vlaev et al., 2011). Neurobiological data can provide converging evidence for this claim by showing that single cells, populations, or brain regions represent reward and other costs together. In a second step the form of neural integration could be determined. As discussed in Section “Using Neurobiological Evidence to Decide between Models of Risk and Delay” the influence of risk and delay on reward has been described in detail, and great strides have been made in our understanding of the neurobiological underpinnings of this process. We know relatively little, however, about how rewards are integrated with other costs.

We begin with a brief review of the regions thought to be involved in this process when outcomes involve a mix of rewarding food and physical effort. The dopamine system and the nucleus accumbens (NAc) are central to animals’ motivation to overcome effort in order to obtain a larger reward (Phillips et al., 2007; Salamone et al., 2007; Floresco et al., 2008a,b). Dopamine antagonists and lesions of the NAc decrease the probability that high-reward/high effort options are chosen, while dopamine agonists make this choice more likely (Cousins and Salamone, 1994; Bardgett et al., 2009; Ghods-Sharifi and Floresco, 2010; Mai et al., 2012). Although exerting effort often takes time and therefore delays reward delivery, there is evidence that the dopamine system and the NAc are important for overcoming effort even when the delay to reward is controlled (Floresco et al., 2008b; Ghods-Sharifi and Floresco, 2010). For example, Day et al. (2011) used voltametry to show that in forced choice trials NAc dopamine release expressed the discounted reward value of future outcomes associated with either effort or delay. When food reward was associated with low effort or delivered immediately, dopamine release was higher than when the same amount of food was associated with high effort or delivered after a longer delay. One difficulty in interpreting these results was that exerting effort inevitably resulted in a time delay between the cue and reward delivery. However, because dopamine release was significantly lower in the high effort relative to the long delay trials the authors could conclude that the attenuation of dopamine release in high effort trials was not solely due to the time delay associated with the effort cost. The role of dopamine is not limited to physical effort but also extends to cognitive effort (Cocker et al., 2012).

The NAc is part of a network of inter-connected regions that play a role in effort-based decision-making which includes the ACC (Walton et al., 2002, 2003; Schweimer and Hauber, 2005; Rudebeck et al., 2006) and basolateral amygdala (Floresco and Ghods-Sharifi, 2007; Ghods-Sharifi et al., 2009). Animals can overcome the effects of ACC lesions with additional training (Rudebeck et al., 2006) or when the ratio between the easy-smaller and the harder-larger rewards increases (Walton et al., 2002), and ACC lesions do not always alter effort-based choices (Schweimer and Hauber, 2005), suggesting that it plays less of a key role than the NAc (Floresco et al., 2008a).

**Figure 5:**
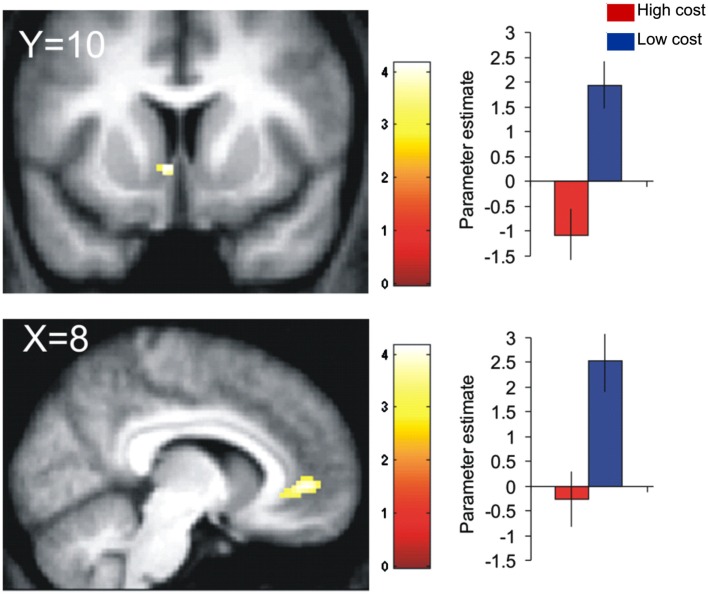
**Neurobiological evidence for the bilinear model**. BOLD signal in the ventral striatum (top) and subgenual cingulate gyrus (bottom) covaried positively with reward in the low-cost conditions (blue), in which participants decided between mixtures of money and mild electric stimulation. This correlation was attenuated in the high-cost condition (red), in which participants decided between mixtures of money and painful electric stimulation. Figure adapted from Talmi et al. (2009).

In line with this animal work, Talmi et al. (2009) and Park et al. (2011) found that a medial PFC region extending from the subgenual/perigenual ACC to vmPFC/OFC expressed the bilinear interaction between reward and pain cost at the time of decision (Eq. 6). Talmi et al. (2009) showed that activation in the subgenual ACC that was parametrically modulated by monetary reward was attenuated when the rewarding outcome also involved pain (Figure 5). Park et al. (2011) replicated these results, demonstrating that pain-discounted values in this region fitted the bilinear model (Eq. 6) better than the additive model (Eq. 1), and even more so when the utility function in Eq. 6 was modeled as a power function. Talmi et al. also observed the same pattern in VS, in the region of the NAc. Park et al. did not find such activation in the VS but reported increased connectivity between the subgenual ACC and the amygdala when outcomes involved high compared to low pain. Notably, the VS and amygdala regions reported by these authors were only 13 mm apart. In summary, these two datasets suggest that the vmPFC and possibly the VS and amygdala express the modulation of reward by pain costs. The convergence on these regions is not surprising given their ubiquitous role in representing subjective value across a variety of paradigms (Levy and Glimcher, 2012). Rather, these studies are important because of their computational approach, which allowed them to demonstrate that neural signals in these regions conformed better to an interactive than an additive valuation of mixed outcomes.

Hare et al. (2008) pointed out that decision values often correlate with the utility of reward *U*(*m*) and with reward prediction errors, and optimized their task to decorrelate these three factors. They observed that the ACC/vmPFC region close to the regions where decision value was expressed in the studies of Talmi et al. (2009) and Park et al. (2011) expressed *U*(*m*), not *V*, casting some doubt on the interpretation of the above findings. This concern is addressed, however, by the pattern of results in Talmi et al.’s study. In that study pain costs significantly interacted with reward value in both ventral ACC and VS, suggesting that this signal does not merely express reward utility.

Amemori and Graybiel (2012) report results that seem at first glance to challenge the bilinear model (Eq. 6). They recorded from pregenual ACC (close to the region that expressed the bilinear interaction in Talmi et al., 2009) when monkeys decided between accepting a minimum amount of food (“avoidance” decisions) and a reward that was paired with an aversive air puff (“approach” decisions). Both food amount and the strength of the air puff were manipulated parametrically. The additive model (Eq. 1) fitted behavioral choice best, better than the interactive model (Eq. 6) and more complex second and third order models.

An additional result from the same study, however, suggests that an additive model may not tell the whole story. Neuronal activity in pregenual ACC when monkeys anticipated mixed outcomes correlated with subjective value, computed according to the winning additive model, with one population of neurons coding *V* positively and the other negatively. Those positive-coding and negative-coding neural populations were, for the most part, evenly intermixed within the recording area, but a subzone in the ventral bank of the cingulate sulcus had a higher concentration of negatively coding neurons. Microstimulation of this subzone increased avoidance behavior, biasing monkeys to forego the mixed outcome (large reward and air puff) in favor of smaller rewards. The authors plotted cost as a function of reward, noting the decision boundary – the mixture of costs and benefits that resulted in indifference. Trials where the stimulation was “on” had shallower indifference function slopes than trials where the stimulation was “off,” indicating reduced sensitivity to reward under stimulation. The anxiolitic drug diazepam abolished this effect of the stimulation.

To interpret these more complex data it may be helpful to consider the individual differences in Talmi et al.’s (2009) dataset. Only about half of their participants exhibited shallower reward sensitivity under pain; the other participants appeared to decide according to an additive model. The former participants, those who exhibited the interaction, likely experienced the pain cost as more threatening, because their SCR responses to pain were higher and they activated the anterior insula – a region associated with the emotional response to pain (Craig, 2003) – more strongly than those whose reward sensitivity did not change. We speculate that microstimulation in Amemori and Graybiel (2012) may have similarly rendered the aversive air puff more threatening for the monkeys, resulting in attenuated reward sensitivity.

A different map of activation altogether was obtained by Prevost et al. (2010). They used an elegant design where delay and effort costs were manipulated as closely as possible using identical trial structures. Subjective value for mixtures of reward and delay or effort was computed according to the hyperbolic model (Eq. 4). Attesting to the effectiveness of their paradigm, their delay data localized subjective value to the VS and vmPFC, replicating commonalities across many previous studies (Levy and Glimcher, 2012), and closely resembling those of another dataset which also employed the same hyperbolic model to model delay-discounted reward (Peters and Buchel, 2010). This makes the dissociation they observed between the representation of subjective value in the delay and the effort condition particularly striking. Prevost et al. reported that effort-discounted value negatively correlated with signal in the dorsal ACC and anterior insula. Signals in these regions increased when outcomes were more effortful and subjectively less valuable.

Croxson et al. (2009) have similarly observed an interaction between effort and reward in the dorsal ACC. Their task employed a forced choice paradigm, allowing a clear distinction between valuation *per se* and decision-making. They were interested in the location of BOLD responses associated with the subjective value of cues that signaled mixtures of reward and effort. The subjective value was modeled according to a variant of the trade-off model in Eq. 3, and correlated with activity in the dorsal ACC, striatum, and midbrain; yet only the dorsal ACC expressed the interaction of effort and reward, while dopaminergic midbrain and VS expressed both reward and effort but not their interaction. The difficulty in relating Prevost et al.’s and Croxson et al.’s datasets to each other is that although both studies observed an interaction between reward and effort in the same region of the ACC, the correlation between that signal and subjective value was negative in the former and positive in the latter study. By contrast, the correlation between that signal and the level of effort required in each trial was positive in the former and negative in the latter study.

While effort is often associated with activation in the ACC, animal data provides less evidence for effort representation in the insula (Floresco et al., 2008a). Yet a relationship between insula activation and value, in the same direction as that reported by Prevost et al., was also observed in two recent studies. Brooks et al. (2010) required participants to decide between a standard delivery of 10 painful shocks and a gamble, in which either more than 10 or less than 10 shocks could be delivered in equal probabilities. As in Prevost et al. (2010) Brooks et al. also reported negative correlations between subjective gamble values and activation in the dorsal ACC and insula. In keeping with the dominant pattern in the literature, however, they also observed a positive correlation between subjective value and activity in VS. In the second study, a PET study with [18F] fallypride and d-amphetamine challenge, individual differences in dopamine function in the bilateral insula were correlated with their tendency to choose to spend more time and exert more effort in order to win larger rewards (Treadway et al., 2012). Participants who were willing to spend more time and effort for larger rewards – those who presumably evaluated this choice to have a higher subjective value than other participants – exhibited reduced dopamine function in the insula. At the same time, in line with the prevalent pattern in the literature, dopamine function in the striatum and vmPFC correlated positively with this individual difference.

The negative correlation of the insula signal with reward in Treadway et al. (2012), Brooks et al. (2010), and Prevost et al. (2010) may be related to the salience of the more effortful trials. Participants in Prevost et al.’s study may have perceived effort but not delay costs to be salient; the more effortful and time-consuming options were also likely more salient to those of Treadway et al.’s participants who chose them only infrequently, and high probability of receiving more painful shocks could also have been more salient. The “salience network” (Seeley et al., 2007), intriguingly, is identified with conjoint activation in the very same regions, dorsal ACC and bilateral insula, observed by Prevost, Brooks, and their colleagues. This reverse-inference does not, however, explain why effort-discounted value in Prevost et al.’s study did not activate the VS and vmPFC, as delay-discounted value did.

Clearly, even if the hyperbolic model does account both for the effect of effort on decision value and for the effect of delay on these values, it does not necessitate that the two share a neurobiological mechanism. A well-known set of studies demonstrated that Marmosets were willing to wait longer than Tamarins for a larger food reward, but preferred a food reward that was closer in distance to food reward that was further from them in spatial distance (Stevens et al., 2005). Because all animals grew up in captivity with limited exposure to predators, the most likely interpretation for the discounting, in Marmosets, of spatially distant food rewards was the energetic cost (effort) involved in obtaining that reward, rather than risk of predation. The double dissociation may suggest separable mechanisms for effort and delay discounting, or it could indicate differences in valuing these two costs upstream to the decision-making process, as per the discussion of *D* and *U* in Section “Evidence for Separate Representations of *D* and *U*.”

The direction of the correlation between outcome mixtures and VS activity is also not without controversy. On the one hand, Kurniawan et al. (2010) also reported positive value coding in VS for mixtures of reward and effort. Although behaviorally, effort and reward did not interact significantly, the fMRI data suggested a neurobiological interaction. NAc activity was positively correlated with reward magnitude, a correlation that was only significant when participants chose to expend effort for large rewards, but not when they chose to expend effort for smaller rewards or when they chose the low-reward, low effort option. On the other hand, Botvinick et al. (2009) observed stronger NAc activation when a cue signaled a more effortful task. One possibility is that because participants were not offered any reward in that study, the direction of value coding in the VS may have reversed; but in Brooks et al. (2010) the choices were also between “bad” and “worse,” and VS activity correlated positively with reward. Interestingly, Botvinick and colleagues interpreted their data according to Kivetz’ model (Eq. 5), suggesting that NAc activation signals the obligatory shift of the reference point of the utility function to the right in effortful blocks.

We have reviewed neurobiological evidence that accords with interactive models of valuation, however the additive model dominates the human imaging literature. To take just one example from a particularly elegant study, Hare et al. (2008) used an additive model (Eq. 1) to compute the decision value, which in their study was the difference between the true value of a food item, established according to the elicited “willingness to pay” for that item in a Becker–DeGroot–Marschak (BDM) auction procedure, and the price at which the food item was offered to the participant. Hare et al. optimized their task to decorrelate this decision value from *U*(*m*) and a reward prediction error signal, and observed a positive correlation between central OFC activity and this decision value (see also Plassmann et al., 2007). Similarly to studies that used only one model to fit their data, or compared the fit only between two models, this converging evidence for the additive model of valuation cannot rule out the possibility that an interaction term would have improved the fit.

In summary, computationally inspired studies of decision valuation, in participants deliberating between mixed outcomes, have produced converging evidence for additive as well as interactive models when correlating the value computed according to these models with neural activity. However, since most neuroimaging studies compare two models at most, it is possible that more convergence could be achieved by greater employment of model comparison.

## Conclusion

The goal of much neurobiological work on valuation is to understand internal reward representations, namely, how the brain represents costs and benefits that are present in the environment (Dayan, 2012). Here we asked how the subjective value of outcomes is established when they consist of mixtures of costs and benefits. This is a surprisingly under-researched topic despite a large empirical and computational body of work on decision-making.

The way people value costs and benefits, individually, has been studied extensively. Here we reviewed current thinking and empirical data concerning the subjective value of monetary gains and losses, and the influence of risk and time delay on this value. We discussed data that support and challenge available models, and the potential for neurobiological work to illuminate some open questions. By comparison, the functional form of cost-benefit analysis – the decision between mixtures of rewards and costs – is relatively unknown. We described two general classes of models – additive and interactive – for the process of integrating rewards and costs into a single decision value. The economic literature typically assumes that costs and benefits are integrated additively, but there is also support for a variety of interactive models. Yet only a handful of studies directly compare additive and interactive models, or between interactive model variants. Modeling-informed empirical work is clearly necessary in order to enhance understanding of the neurobiological mechanism that allows animals and humans to integrate costs and benefits. Empirical work on this intriguing question should proceed with caution; not assuming that integrated representations of the subjective value of anticipated outcomes are natural kinds, but to demonstrate their existence empirically (Vlaev et al., 2011). We hope that by clarifying some of the main candidates for valuation, and the way neurobiological data can support or challenge them, we will encourage further empirical work on the mechanism that allows animals and humans to decide optimally in a complex environment.

## Conflict of Interest Statement

The authors declare that the research was conducted in the absence of any commercial or financial relationships that could be construed as a potential conflict of interest.

## Footnotes

^1^Peters and Buchel included magnitude (*m*), probability (*p*) and the inverse of delay (1/*d*) as regressors in their model. These regressors were orthogonalised with respect to the regressor that coded value (*V*). Crucially, because *m*, *d* and *p* would, to some extent, be correlated with *V*, and because the analysis model ensured that *V* was assigned all variance that correlated with it, it is difficult to ascertain which of the regions that seemingly coded *V* actually coded *m*, *d* or *P*. This perspective may explain why the authors obtained a different map of activation when they reversed the order of orthogonalization.

## Appendix

Phillips et al. (2007) suggested that when a participant is faced with a choice of taking or foregoing an action which would yield both rewards *M*_R_ and costs *M*_C_, the value of the action can be expressed as the value *V*(*M*_R_, *M*_C_) relative to the value of the same action if cost was not involved, *V*(*M*_R_, *M*_C_ = 0). When there are no costs the value is maximal because *V*(*M*_R_, *M*_C_) = *V*(*M*_R_, *M*_C_ = 0).

They observed that there is a specific cost *C*_i_ which translates to *V*(*M*_R_, *M*_C_) = 0, namely, lead the participant to be indifferent about the choice. When *M*_C_ = *C*_i_ participants are not motivated either to act or avoid acting; consequently, they act 50% of the time. Costs higher than *C*_i_ mean that *V*(*M*_R_,*M*_C_) < 0 and bias the participant against the action; costs lower than *C*_i_ mean that *V*(*M*_R_,*M*_C_) > 0 and favor taking the action. The authors note that there is limited evidence as to the exact functional forms of the indifference function *C*_i_ and offered a tentative, plausible form in their Figure 1, where cost is a linear function of dopamine:

(A1) $$M_{C}=a_{1}\times \text{DA}$$

With the constant *a*_1_ > 0. Given evidence that dopamine level (DA) is a function of currently available reward and the maximum levels of dopamine DAmax observed in the task context, and that the utility of reward is a decelerating function of reward magnitude, DA was described according to the following function:

(A2) $$\text{DA}=\text{D}{\text{A}}_{\max}\times \frac{M_{R}}{\left(M_{R}+a_{2}\right)}$$

With the constant *a*_2_ > 0. Therefore,

(A3) $$C_{i}=a_{3}\times \frac{M_{R}}{\left(M_{R}\text{ + }a_{\text{2}}\right)}$$

With the constant *a*_3_ > 0.

The authors further proposed a specific form for a cost-benefit function *Z* (Figure 2), which depicts the ratio of outcome with cost to the same outcome without cost.

(A4) $$Z=\frac{V\left(M_{R},M_{C}\right)}{V\left(M_{R},M_{C}=0\right)}.$$

Because when there is no response cost the two outcomes are equivalent (the ratio is maximal), and because when the ratio is 0.5 decision-makers are indifferent between the two outcomes, we determine that *Z* passes between (*C*_i_, 0) and (0,1). Therefore

(A5) $$Z=\frac{1-M_{C}}{C_{i}}$$

The authors did not provide an explicit model for *V*(*M*_R_, *M*_C_ = 0), the utility of rewarding outcomes that are not accompanied by costs. On the basis of their choice of Eq. 2 to represent DA and their reasoning for that choice we selected the same form to also model reward utility:

(A6) $$V\left(M_{R},M_{C}=0\right)=R_{\max}\times \frac{M_{R}}{\left(M_{R}+a_{4}\right)}$$

Combining the above equations provides us with a value function for mixed outcomes:

(A7) $$\begin{array}{c} V\left(M_{R},M_{C}\right)=\left[R_{\max}\times \frac{M_{R}}{\left(M_{R}+a_{4}\right)}\right]-\left[\left(\frac{R_{\max}}{a_{3}}\right)\times {\text{M}}_{C}\times \frac{\left({\text{M}}_{R}+a_{\text{2}}\right)}{\left({\text{M}}_{R}+a_{\text{4}}\right)}\right] \end{array}$$

To understand its implications we considered the case where Mr < < *a*4, namely when the utility of Mr, *V*(*M*_R_, *M*_C_ = 0), resembles a linear function. In this case, some algebra will show that

(A8) $$V\left(M_{R},M_{C}\right)=\left(\frac{R_{\max}}{a_{4}}\right)\times \left({\text{M}}_{\text{R}}-\left[\left(\frac{{\text{a}}_{1}}{{\text{A}}_{\text{3}}}\right)\times M_{C}\right]-\left[\left({\text{M}}_{R}\times \frac{M_{C}}{a_{\text{3}}}\right)\left(1-\frac{a_{2}}{a_{4}}\right)\right]-\left[\frac{R^{2}}{a_{4}}\times \left(1-\frac{M_{C}}{a_{\text{3}}}\right)\right]\right)$$

Now, as M_R_ < < *a*_4_, we could probably drop the last term as it is small (it is always a positive term) and “tidy up,” introducing constants *x*_1_, *x*_2_, and *x*_3_’:

(A9) $$V\left(M_{R},M_{C}\right)=X_{1}\times {\text{M}}_{R}-X_{\text{2}}\times M_{\text{C}}-X_{\text{3}}\times {\text{M}}_{R}\times M_{\text{C}}$$

With *x*_3_ a constant that is either positive or negative:

(A10) $$X_{3}=\left(\frac{R_{\max}}{a_{4}}\right)\times \frac{\left(\frac{1-a_{2}}{a_{\text{4}}}\right)}{a_{3}}$$

And *x*_1_ and *x*_2_ positive constants.
